# Treatment effect of mTOR-inhibition on tissue composition of renal angiomyolipomas in tuberous sclerosis complex (TSC)

**DOI:** 10.1371/journal.pone.0189132

**Published:** 2017-12-12

**Authors:** Susanne Brakemeier, Lars Vogt, Lisa Adams, Bianca Zukunft, Gerd Diederichs, Bernd Hamm, Klemens Budde, Marcus R. Makowski

**Affiliations:** 1 Department of Nephrology and Medical Intensive Care, Charité, Berlin, Germany; 2 Department of Radiology, Charité, Berlin, Germany; 3 King’s College London, Division of Imaging Sciences, London, United Kingdom; National Cancer Institute, UNITED STATES

## Abstract

**Purpose:**

Tuberous sclerosis complex (TSC)-associated renal angiomyolipoma (AML) have a high lifetime risk of acute bleeding. MTOR-inhibitors are a promising novel treatment for TSC-AML, however adequate response to therapy can be difficult to assess. Early changes in MRI signal may serve as a novel early indicator for a satisfactory response to mTOR-inhibitor therapy of AML.

**Materials and methods:**

Thirty-eight patients with the definite diagnosis of tuberous sclerosis receiving everolimus therapy and n = 19 patients without specific therapy were included. 1.5 Tesla MRI was performed including sequences with a selective fat suppression. Patients were investigated prior to the initiation of therapy (baseline) and after <3 months (n = 21 patients), 3 to 6 months (n = 32) and 18 to 24 months (n = 28). Signal and size changes of renal AMLs were assessed at all different timepoints. Signal-to-noise-ratio (SNR), contrast-to-noise-ratio (CNR) and size of angiomyolipomas were evaluated.

**Results:**

Signal changes in 273 AMLs were evaluated. A significant and strong decrease of the CNR of AMLs following the initiation of therapy was measured in the fat-suppressed MR sequence at all time points, compared to the baseline: From 7.41±6.98 to 3.84±6.25 (p ≤ 0.05p = 0.002), 3.36±6.93 (p<0.0001), and 2.50±6.68 (p<0.0001) after less than 3 months, 3–6 months or 18–24 months of everolimus treatment, respectively. Also, a significant, however less pronounced, reduction of angiomyolipoma size in the different groups was measured (from baseline 2022.2±2657.7 mm2 to 1854.4±1670.9 mm2 (p = 0.009), 1875.5±3190.1 mm2 (p<0.001), and 1365.8 ± 1628.8 mm2 (p<0.0001) after less than 3 months, 3–6 months or 18–24 months of everolimus treatment, respectively). No significant changes in CNR (p>0.05) and size (p>0.05) were measured in the control group.

**Conclusion:**

mTOR inhibitor therapy in TSC patients results in an early and pronounced fatty transformation of AMLs on MRI. Fatty transformation could represent a novel early indicator of response to therapy in this patient collective.

## Introduction

Tuberous sclerosis complex (TSC) is a rare autosomal dominant disorder that affects approximately 1.5 million people worldwide with a birth incidence of 1 in 6000 [1]. More than 70% of patients present with a sporadic genetic mutation and have no family history of TSC [2]. The clinical picture of TSC is characterized by the proliferation of different types of hamartomas in various organ systems, including the kidneys, brain and skin. Renal angiomyolipomas (AMLs) are observed in more than 80% of patients with TSC. Starting in late childhood, multiple AMLs manifest bilaterally being associated with a size-related risk of acute bleeding. As a consequence, renal complications are the leading cause of death in adult TSC patients [3–5] and AML-related surgery is performed in a high percentage of adult TSC patients [6]. Additionally, the continuous increase in angiomyolipoma size can lead to a compression of remaining healthy kidney tissue increasing the risk for development of chronic kidney disease.

TSC is caused by decreased or absent expression of the genes TSC1 (hamartin) or TSC2 (tuberin) resulting in an aberrant mTOR-signaling and subsequent tumor growth [7]. The mTOR-inhibitor everolimus has been approved for the treatment of TSC-AML, as a significant overall reduction in AML size could be demonstrated in a prospective randomized study [7]. An overall reduction of more than 50% of the total volume of AMLs relative to the baseline was chosen as criterion for a favourable response in this study [7]. Such a high cutoff value to assess response to therapy had to be chosen, as the precise assessment of size changes in angiomyolipomas can be challenging due to the heterogeneity of angiomyolipomas and spontaneous changes in morphology resulting from e.g. focal bleedings. Therefore, additional early indicators to assess the response to mTOR inhibitor therapy would be helpful for the clinical management of a high number of TSC patients.

Beyond that, it has not been investigated how mTOR inhibitor therapy affects the different tissue types of angiomyolipoma, vascularization (angio-), myocytes (-myo-) and lipid cells (-lipoma), during the time-course of therapy.

The aim of this study was to evaluate potential changes in the relative tissue composition of renal angiomyolipomas following the initiation of mTOR inhibitor therapy based on MRI measurements. Additionally, the time course of changes was evaluated.

## Material and methods

### Study population

All patients were older than 18 years and definite diagnosis of TSC was established based on current diagnostic guidelines [2]. We herewith state that all data were anonymized before access by the researchers. The Charité ethics committee approved our retrospective study and waived the requirement for informed consent.

Patients without a definite diagnosis of TSC were excluded from the analysis. MRI was not performed in patients with standard contraindications for MRI, including claustrophobia, specific metallic—items such as cochlear implants, central nervous system aneurysm clips, pacemakers/ defibrillators). Additionally, MRI data sets of the abdomen/kidneys had to be available prior to and following the initiation of the mTOR inhibitor therapy.

Overall, a study population of 38 TSC patients with 273 angiomyolipomas and MR examination before initiation of everolimus treatment were included in this study. Of these, 21 patients (13 female patients, age 44.3 ± 10.8 years, 57 angiomyolipomas) was imaged within three months of the initiation of the everolimus therapy, a group of 32 patients (20 female patients, age 39.7 ± 11.6 years, 89 angiomyolipomas) was imaged within 3 to 6 months of initiation of the everolimus therapy, a group of 27 patients (17 female patients, age 41.8 ± 12.1 years, 77 angiomyolipomas) was imaged within 18 to 24 months of initiation of the everolimus therapy. Antiepileptic co-medication was recorded and everolimus dosing as well as trough levels at time of MRI imaging were documented. Adverse events of grade 2 or higher according to MedDRA version 12.1 were recorded as well as everolimus treatment interruptions for longer than 2 weeks.

Additionally, a control collective of 19 TSC patients (15 female patients, age 39.2 ± 12.1 years, 50 angiomyolipomas) without mTOR inhibitor therapy and solely follow-up MR examinations was included. For details regarding the TSC patient population please see Table 1.

**Table 1:** Characteristics of the investigated TSC patient cohort.

	Overall population	Time period between baseline and follow-up (months)			
		0–3	3–6	18–24	control
AML number	273	57	89	77	50
Female (%)	25 (65.8)	13 (61.9)	20 (62.5)	17 (63.0)	15 (78.9)
Male (%)	13 (34.2)	8 (38.1)	12 (37.5)	10 (37.0)	4 (21.1)
Age (years)	38.2 ± 11.5	44.3 ± 10.8	39.7 ± 11.6	41.8 ± 12.1	39.2 ± 12.1

Patients were investigated at baseline and after 0–3 months, 3–6 months and 18–24 months of mTOR inhibitor therapy. Additionally, a control collective of TSC patients without mTOR inhibitor therapy was investigated. The time period between baseline and follow-up scan was 9.50 ± 10.0 months in the control collective.

### MR imaging protocol

MRI imaging data sets were acquired between January 2010 and January 2017. MRI imaging was performed on a 1.5 T system (Avanto; Siemens Medical Solutions, Erlangen, Germany) equipped with a standard abdomen matrix coil. First a T2 haste non-breathhold localizer scan was performed for anatomical orientation. Based on this scan the following sequences were planned: Transversal T1 spin echo and the following parameters: Field of view 320 mm, and matrix 256, slice thickness 5 mm, TR 550 ms, TE 8.4 ms, to averages, flip angle 90°. Transversal T2 haste: Field of view 320 mm, matrix 320 slice thickness 4 mm, TR 800 ms, TE 94 ms, averages 1, flip angle 180°. Coronal T2 haste: Field of view 400 mm, matrix 320, slice thickness 5 mm, TR 800 ms, TE 89 ms, averages 1. Transversal T1 flash (including in phase and opposed phase): On field of view 400 mm, matrix 320, slice thickness 4 mm, averages 1. Additional sequences included: Non-breathhold T2 haste scout to plan the following transversal free breathing navigator-gated T2 fatsat sequence (T2 FS) turbo spin echo (TSE) sequences, field of view 320 mm, matrix 320 slice thickness 4 mm, TR 3300 ms, TE 79 ms, 2 averages, flip angle 180°, frequency selective fat saturation.

### Imaging analysis

All images were analyzed using PACS workstations (Centricity Radiology RA1000; GE Healthcare, Little Chalfont, United Kingdom). Images were analysed independently, randomized and blinded to all clinical information.

All imaging analysis was based on the navigator-gated T2 fatsat sequence (T2 FS) which was available in all patients. The main advantage of this sequence was, that it could be acquired in free breathing, compared to the other MR sequences of the MR imaging protocol which were breathhold sequences. Therefore, AMLs could be clearly delineated with a high spatial resolution. Breathing artifacts resulting from breath-hold artifacts were not present. This is especially relevant in patients with TSC-related developmental delays.

In all patients, the largest three AMLs where evaluated and the area in mm2 was recorded. Measurements based on multiple regions of interests (ROIs) were performed on the imaging slice which demonstrated the largest angiomyolipoma area. Such an approach was chosen, as this represents a typical clinical approach for the assessment of the size of AMLs.

Additionally, signal measurements of the angiomyolipomas were performed based on the same regions of interest as the size measurements. This included the assessment of the SNR (signal-to-noise-ratio) and CNR (contrast-to-noise-ratio) values of all angiomyolipomas. For the calculation of the SNR and CNR the following signal intensities were measured. The signal of the angiomyolipoma (SI_AML_), the signal of a reference muscle (SI_muscle_) and the noise (Signal_N_). Noise (N) was determined in a ROI placed in the air ventral to the abdomen. The signal-to-noise-ratio (SNR) was measured between AMLs (SI_AML_) and noise (Signal_N_.): SNR = Signal_AML_ / Signal_N_.

Contrast-to-noise-ratio (CNR) was measured between AMLs (SI_AML_) and muscle tissue (SI_muscle_). The CNR was calculated by the following formula:

$$\text{CNR}=\left({\text{Signal}}_{\text{AML}}-{\text{Signal}}_{\text{muscle}}\right)/{\text{Signal}}_{\text{N}}.$$

### Statistical analysis

The Shapiro-Wilk-Test was used to test for normal distribution. To determine whether covariates such as age/sex had an influence on the mean values and their differences, Kendall’s-tau-b correlations were performed. To test for significance between baseline and follow-up measurements linear mixed model analysis was performed.

Variables are reported as mean ± standard deviation. A p-value < 0.05 was considered statistically significant. Statistical analyses were performed using SPSS for Windows release 24.0.0 (SPSS Inc., Chicago, IL, USA).

## Results

### Signal-to-noise-ratio and contrast-to-noise-ratio measurements of angiomyolipomas in TSC patients following mTOR inhibitor therapy

**Fig 1:**
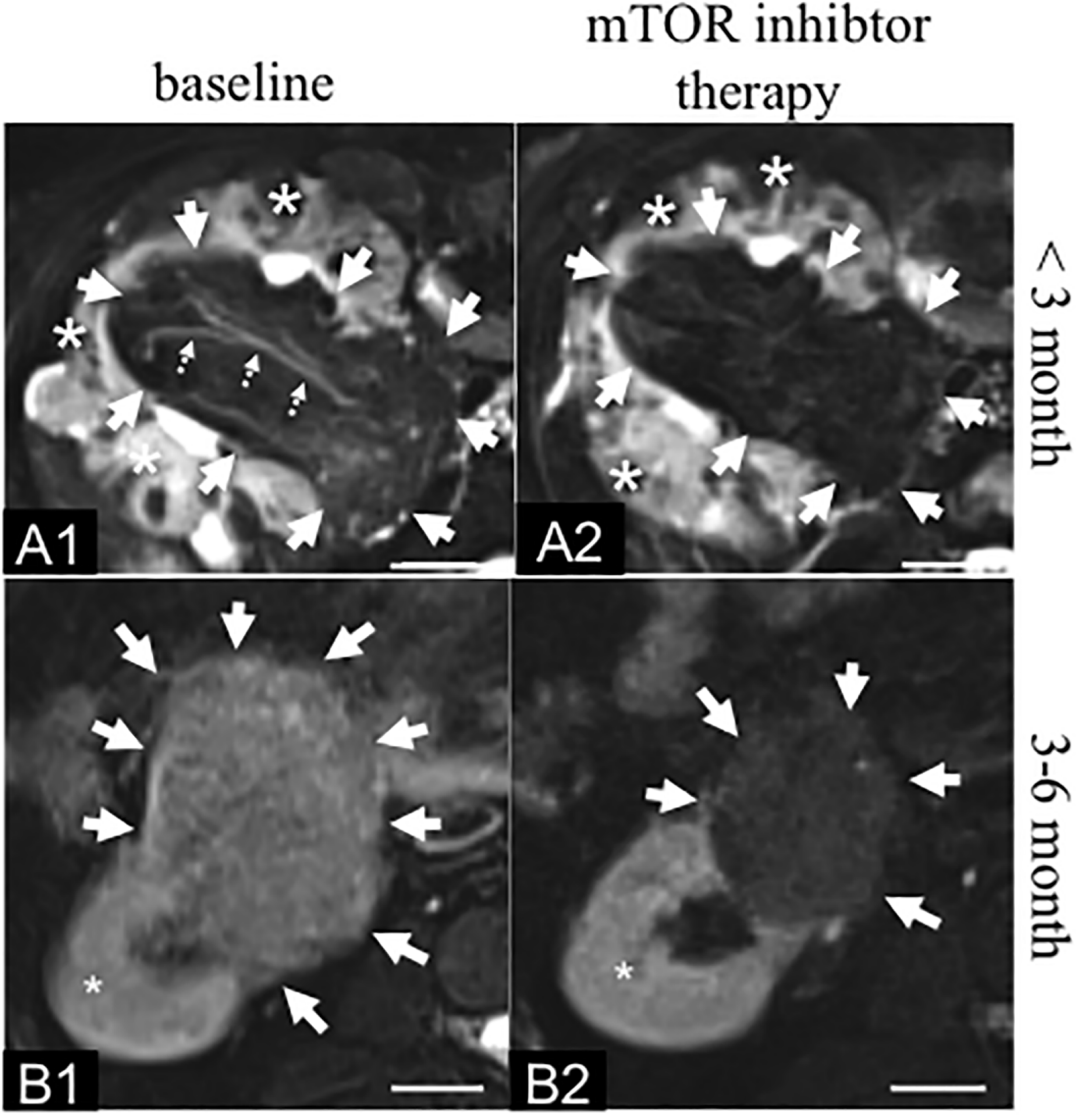
Fatty transformation of angiomyolipoma in TSC patients after initiation of mTOR inhibitor therapy. **A**: Example of a large AML at the central part of the right kidney in a 33 year-old female TSC patient. **A1**: At baseline a heterogeneous AML with a mixed-signal on the T2 fat saturated MR sequence (solid arrows) could be visualized. Slow flowing blood in a small vessel (dotted arrows) appears hyperintense on the fat saturated sequence. The unaffected “healthy” kidney tissue is also visualized with a hyperintense signal (*). **A2**: 2.5 months following the initiation of the mTOR inhibitor therapy a reduction in the overall signal of the AML on the T2 fat saturated sequence can be visualized. The vessel, which was visible at baseline (A1: dotted line) cannot be delineated anymore. Additionally, a reduction of size of the AML can be visualized. This case represents a good example of how difficult it can be to clearly quantify a size reduction in a heterogenous AML following the initiation of mTOR therapy. **B**: Example of a AML at the caudal part of the right kidney in a 45 year-old male TSC patient. **B1**: At baseline a heterogeneous relatively bright angiomyolipoma could be visualized on the T2 fat saturated sequence (arrows). The unaffected “healthy” kidney tissue is visualized with a homogenous hyperintense signal on the T2 fat saturated sequence (*). **B2**: 3 to 6 months following the initiation of the mTOR inhibitor therapy, a clear reduction in the signal on the T2 fat saturated sequence as well as in the size of the angiomyolipoma is visualized. The signal of the healthy kidney tissue does not change following the initiation of the therapy (*). MRI: Magnetic Resonance Imaging. Fatsat: Fat saturated. Scale bar: 1.5 cm.

All signal measurements were performed based on ROIs on the navigated T2 sequences in combination with a specific fat saturation pulse (Fig 1).

At baseline, angiomyolipomas showed heterogenous signal properties on the T2 FS MR sequences. A mixture of areas with high and low signals on this sequence were present in all angiomyolipomas (Fig 1). Visually, a strong a decrease in signal was visible on the T2 FS sequence in all follow-up scans compared to the baseline. In the follow-up scans angiomyolipomas appeared relatively homogeneous with a dark signal on the T2 FS sequence (Fig 1).

The baseline SNR and CNR values of angiomyolipomas in the control group were 15.8±6.44 and 8.94±6.07. The time period between baseline and follow-up scan was 9.50 ± 10.0 months in controls. These values did not differ (p > 0.05) from the baseline values of the therapy group.

SNR and CNR were determined at different timepoints following the initiation of the mTOR therapy. Compared to baseline, a significant (p ≤ 0.05) reduction in SNR and CNR was measured already in the group with less than three months of mTOR inhibitor therapy. SNR values demonstrated a reduction from 15.2±7.95 to 11.1±6.85 (p<0.0001), 12.0±6.87 (p = 0.001), and 11.3±7.38 (p = 0.003) after less than 3 months, 3–6 months or 18–24 months of everolimus treatment, respectively.

**Fig 2:**
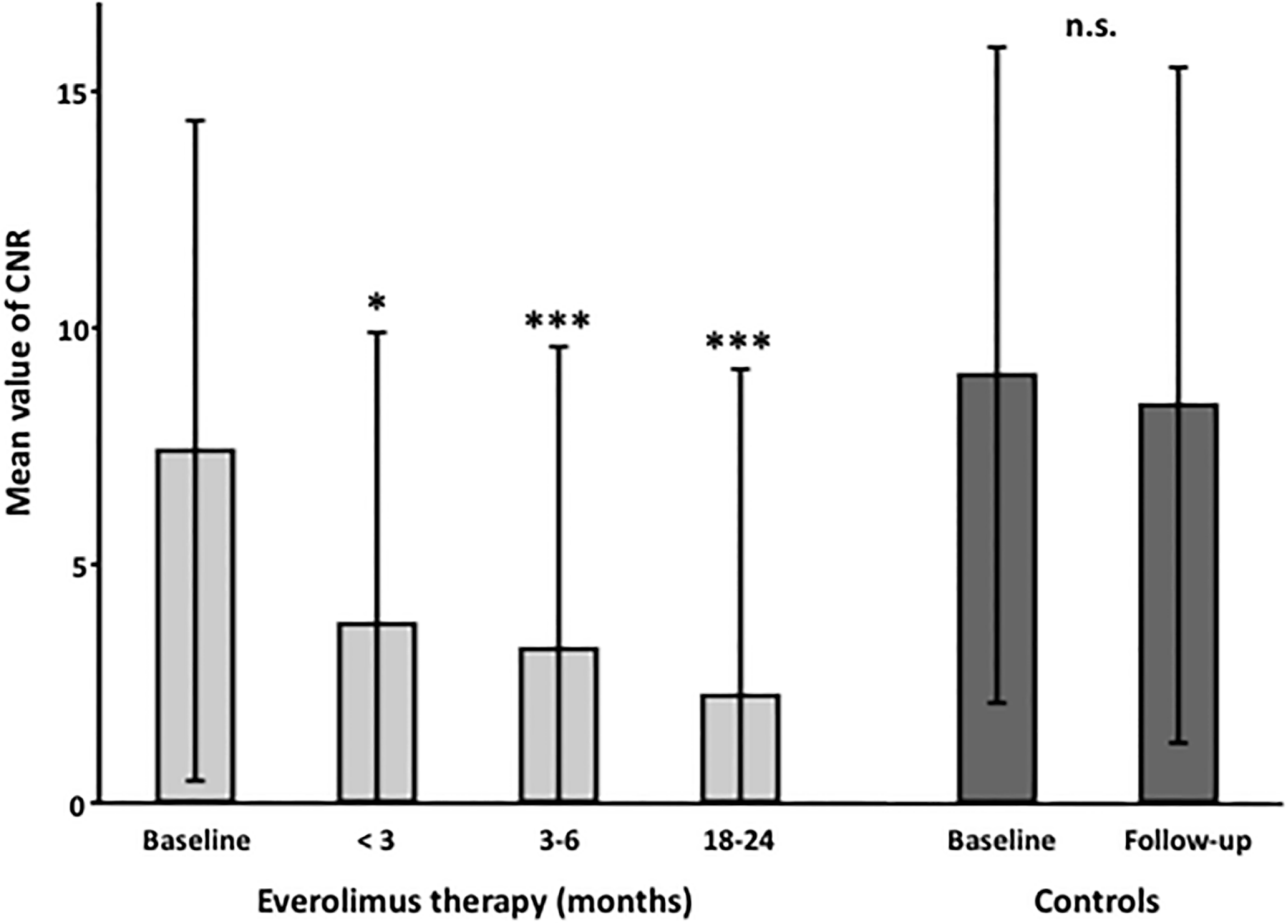
Signal changes in magnetic resonance imaging with selective fat suppression in TSC patients following the initiation of mTOR inhibitor therapy. Patients were investigated at baseline and after 0–3 months, 3–6 months and 18–24 months of mTOR inhibitor therapy. Additionally, a control collective of TSC patients without mTOR inhibitor therapy was investigated. A pronounced and significant reduction of the mean contrast to noise ratio (CNR) was already observed in the early group within the first three months. In the control group, no reduction of the mean CNR value was observed. Error bars indicate the standard deviation. P-values generated from linear mixed model statistical analysis (* p<0.01, *** p<0.0001); CNR: Contrast to noise ratio.

Changes were more pronounced for the CNR measurements. A strong reduction of CNR values from 7.41±6.98 to 3.84±6.25 (p = 0.002), 3.36±6.93 (p<0.0001), and 2.50±6.68 (p<0.0001) was measured after less than 3 months, 3–6 months or 18–24 months of everolimus treatment, respectively (Fig 2).

### Size measurements of angiomyolipomas in TSC patients following mTOR inhibitor therapy

All size measurements were performed based on the same ROIs as the signal to noise ratio and contrast to noise ratio measurements.

In the control group angiomyolipomas demonstrated an average size of 1105.7±1378.4 mm2.

**Fig 3:**
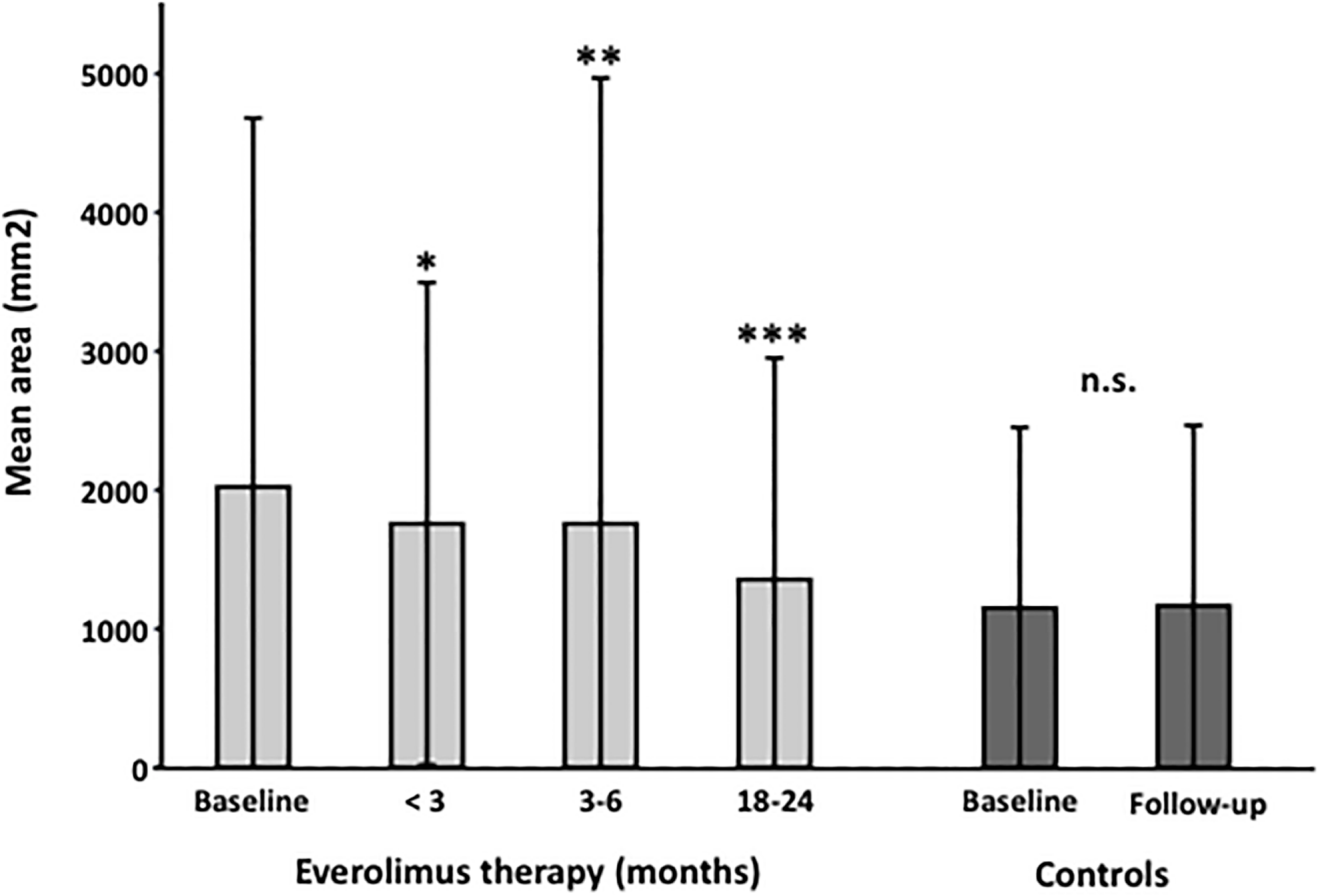
Size changes in magnetic resonance imaging in TSC patients following the initiation of mTOR inhibitor therapy. Patients were investigated at baseline and after 0–3 months, 3–6 months and 18–24 months of mTOR inhibitor therapy. Additionally, a control collective of TSC patients without mTOR inhibitor therapy was investigated. A significant (p ≤ 0.05) reduction of the size of angiomyolipomas (in mm^2^) was already observed in the early group within the first three months. In the control group, no reduction of the size was observed. Error bars indicate the standard deviation. P-values generated from linear mixed model statistical analysis (* p<0.01, ** p<0.001, *** p<0.0001).

Compared to baseline, a significant reduction in angiomyolipoma size was already detected in the group with less than three months of mTOR inhibitor therapy. The size of angiomyolipomas was reduced from 2022.2±2657.7 mm2 to 1854.4±1670.9 mm2 (p = 0.009), 1875.5±3190.1 mm2 (p<0.001), and 1365.8 ± 1628.8 mm2 (p<0.0001) after less than 3 months, 3–6 months or 18–24 months of everolimus treatment, respectively. (Fig 3).

### Relative signal and size measurements of angiomyolipomas in TSC patients following mTOR inhibitor therapy

Relative changes (%) of CNR measurements and changes in size of angiomyolipomas were directly compared. Changes in both parameters occurred simultaneously. Already after three months of mTOR inhibitor therapy, a relative reduction in CNR and area measurements was detected. The average reduction in CNR was however more pronounced (more than 60%) compared to the area measurements (more than 15%). Comparable relative changes in signal reduction were also measured in the 3 to 6 months group and the 18 to 24 months group. This indicates that main treatment effects of mTOR inhibitor therapy seem to occur in the first three months of therapy and only minor further changes occur at later time points. As expected, no significant changes in relative reduction of CNR and area measurements were detected in the control group of patients without mTOR inhibitor therapy.

A relative reduction in CNR of ≥30% were seen in 66.7%; 74.3%, and 92.1% of treated patients after 3 months, 3–6 months, or 18–24 months, respectively. A relative reduction in CNR of ≥50% were seen in 52.1%; 44.1%, and 88.9% after 3 months, 3–6 months, or 18–24 months, respectively.

**Fig 4:**
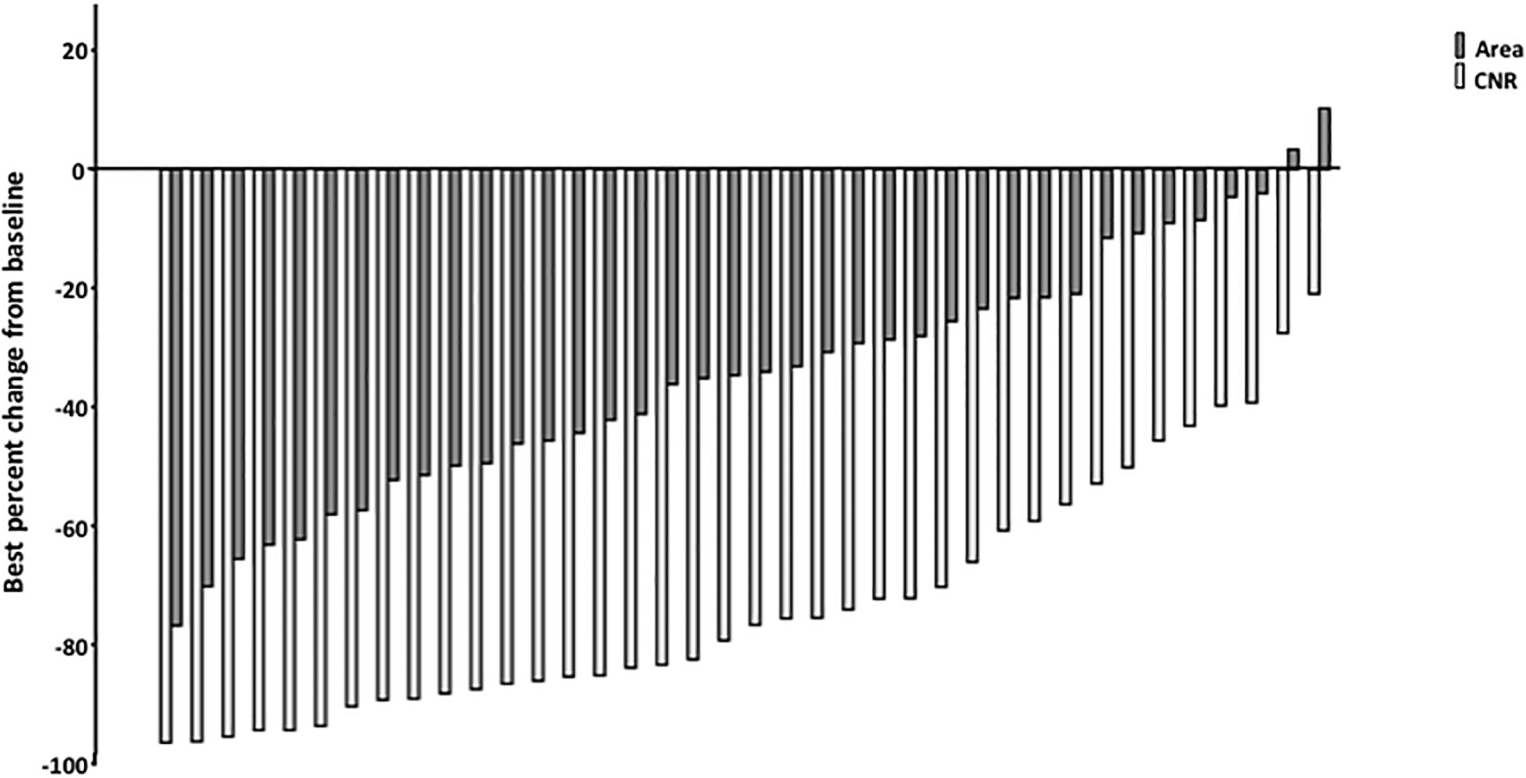
Best percentage reduction of contrast to noise ratio as well as size of angiomyolipomas in each individual patient reported at any time point. In all analyzed patients a reduction in CNR could be observed whereas 2 patients did not show a size reduction of AML under everolimus treatment. AML size is given in mm^2^, results are sorted by value.

Best percentage reduction of CNR and AML area reported at any time point under everolimus therapy is shown in Fig 4.

### Clinical outcome

Dosing and trough levels of everolimus are given in Table 2. AE’s were retrospectively analyzed for the observation period and were in concordance to previously published studies (Table 3) (6,7). All patients presented with measurable trough levels of everolimus at time of MRI analysis, however, dose interruption for longer than 2 weeks was performed in 1 patient during the first 3 treatment months, in 2 patients during month 3- month 6 and in 8 patients during month 18-month 24. In three patients, dose interruption was associated with non-compliance.

**Table 2:** Trough levels and dosing of everolimus.

	Trough level (ng/ml)	Daily dose (mg)
<**3 months, mean ±SD**		
Total cohort	7.83±2.9	8.33±2.4
EIAED+	7.95±4.9	9.38±1.8
EIAED-	7.78±2.6	7.69±2.6
**3–6 months, mean ±SD**		
Total cohort	6.99±4.1	5.98±2.7
EIAED+	6.18±4.4	6.13±2.5
EIAED-	7.57±3.9	5.85±2.9
**18–24 months, mean ±SD**		
Total cohort	6.56±4.9	4.90±2.2
EIAED+	7.07±6.1	5.79±2.7
EIAED-	6.15±2.9	4.13±1.3

EIAED+: subgroup of patients with enzyme-inducing antiepileptic drug use

EIAED-: subgroup of patients without enzyme-inducing antiepileptic drug use

Everolimus trough levels were documented at time of MRI analysis.

**Table 3:** Summary of adverse events of grade ≥2 under everolimus treatment.

Grade ≥2 AEs	Events, n (%)
Aphtous stomatitis	17 (44.7)
Bacterial Infection	14 (36.9)
Hypercholesterinemia	13 (34.2)
Amenorrhoea	7 (33.3)*
Proteinuria	10 (26.3)
Viral Infection	9 (23.7)
Ovarian cysts	4 (16.0)*
Anemia	5 (13.2)
Acne	5 (13.2)
Hypertension	4 (10.5)

* calculated for patients at risk.

No AML bleeding events were documented during the study. Renal function was normal and remained stable in 32/38 (84.2%) of patients receiving everolimus. In 6/38 (15.8%) of treated patients renal function was already impaired at beginning of treatment and further declined from 1.63±0.63mg/dl at baseline to 2.53±1.01mg/dl serum creatinine after 36 months. AML signaling responses were independent of renal function.

In all 19 control patients, renal function was normal and remained stable throughout the observation period.

## Discussion

This study demonstrated that everolimus therapy in TSC patients results in an early and pronounced changes of tissue composition of angiomyolipomas on MRI. The appearance of AMLs in MRI was associated with a rapidly decreasing vascularization of angiomyolipomas and could represent a novel early indicator of response to mTOR inhibitor therapy in this patient collective.

Following the onset of everolimus therapy, changes in tissue composition and size of angiomyolipomas occurred relatively early and simultaneously and were most pronounced in the first three months following the initiation of the therapy. No relevant further therapeutic effects were measured at later time points (3 to 6 months, 18 to 24 months). After three months of therapy, the relative changes regarding the predominance of fatty tissue and the reduction of the vascular component were more pronounced compared to changes in size of angiomyolipomas. An early indicator of response to therapy, besides size, could be particularly important as the exact size of especially large and heterogenous angiomyolipomas can be difficult to assess.

In our study we could detect a treatment response in MRI signaling in every patient receiving everolimus. This is in contrast to the randomized EXIST-2 study analyzing the volume reduction of AML under everolimus treatment where 15% of patients could either not be evaluated or did not show a treatment response [8]. As a consequence, changes in MRI signaling could be more sensitive in detecting AML treatment responses in TSC patients.

Different studies demonstrated that the bleeding risk of angiomyolipomas is directly associated with the size of the angiomyolipoma. Therefore, a clinical cutoff of AMLs larger than 4 cm in diameter was defined for initiation of everolimus therapy [9,10]. For small (< 4 cm) asymptomatic angiomyolipomas, yearly follow-up investigations are recommended. Until now, it has not been investigated whether bleeding risk is different in AMLs consisting almost exclusively of fat and highly vascularized AMLs. It is thought that bleeding risk depends on vascular aneurysms, which are predominantly found in highly vascularized AMLs

If an AML size reduction cannot be achieved or reliably measured after initiation of everolimus therapy the clinical consequence would be dose escalation of everolimus or even AML embolization. The former is associated with a higher rate of side effects, the later with a loss of functioning kidney tissue and potential loss of GFR. An early indicator for response to therapy could help reduce the percentage of uncertain assessments.

On MRI, the different tissue types of angiomyolipomas, mature adipose tissue, dysmorphic blood vessels/microvessels and smooth muscle-like cells show different signal properties. Adipose tissue is hyperintense on T1 and T2 sequences, however hypointense if fat suppression is applied. Smooth muscle cells are T1 and T2 hypointense and do not show a signal loss on fat-suppressed sequences [11,12]. Fluids, tissue edema or slow flowing blood in microvessels appear hyperintense on T2 sequences with fat suppression. It can therefore be assumed that the hyperintense signal on the T2 fat-suppressed sequence, which was detected at baseline in all patient groups and the control group, reflects the vascular component of angiomyolipomas, with slow flowing blood in small angiogenic vessels (microvessels) which developed with the AML.

Further information regarding the tissue composition of AMLs can be derived, if gadolinium based contrast agents are administered. These contrast agents should however be avoided, especially in patients with impaired renal function, which is frequently observed in TSC patients.

In the current study, a pronounced drop in contrast to noise ratio of AMLs was observed in the first three months following the initiation of the mTOR inhibitor therapy. This drop in signal reflected a fatty transformation in combination with a reduction of the vascular/microvascular component of angiomyolipomas. The reduction in the vascular component, which include small angiogenic vessels, could represent the initiating treatment effect of mTOR inhibitors which subsequently leads to a reduction in angiomyolipoma size The reduction in vascularization may be the main effect for effective bleeding prevention, and therefore may represent the true therapeutic effect. The current study is the first study to report such an early effect of mTOR therapy.

This observation is in line with previous studies which demonstrated antiangiogenic properties of everolimus using blood-based biomarkers. mTOR is assumed to play a critical role in tumor vascularization, as it is part of the phosphatidylinositol 3-kinase/Akt/mTOR signalling pathway, which is implicated in tumor angiogenesis [13]. Previous studies in TSC patients indicated that blood levels of tumor vascularization mediators are reduced in response to mTOR inhibitor therapy reflecting the strong effect on AML vascularization [7,14,15].

Limitations of this study: Only adult patients were included in our study. No histopathological validation of changes of the tissue components of angiomyolipoma was performed.

## Conclusion

The precise assessment of size changes in angiomyolipomas can be challenging due to the heterogeneity of these complex lesions and spontaneous changes in angiomyolipomas morphology resulting from e.g. focal bleedings. Fatty transformation of renal angiomyolipoma on MRI could represent a novel early indicator to identify TSC patients which respond to mTOR therapy. Unnecessary dose escalation or even interventional therapy, such as embolization, could possibly be avoided in patients without clear size effect of mTOR therapy.

## References

[1] Budde K, Gaedeke J. Tuberous sclerosis complex-associated angiomyolipomas: focus on mTOR inhibition. Am J Kidney Dis 2012; 59:276–283. doi: 10.1053/j.ajkd.2011.10.013 22130643 10.1053/j.ajkd.2011.10.013

[2] Northrup H, Krueger DA. Tuberous sclerosis complex diagnostic criteria update: recommendations of the 2012 Iinternational Tuberous Sclerosis Complex Consensus Conference. Pediatr Neurol 2013; 49:243–254. doi: 10.1016/j.pediatrneurol.2013.08.001 24053982 10.1016/j.pediatrneurol.2013.08.001 PMC4080684

[3] Henske EP, Scheithauer BW, Short MP, Wollmann R, Nahmias J, Hornigold N, et al Allelic loss is frequent in tuberous sclerosis kidney lesions but rare in brain lesions. Am J Hum Genet 1996; 59:400–406. 8755927 PMC1914733

[4] Franz DN, Bissler JJ, McCormack FX. Tuberous sclerosis complex: neurological, renal and pulmonary manifestations. Neuropediatrics 2010; 41:199–208. doi: 10.1055/s-0030-1269906 21210335 10.1055/s-0030-1269906 PMC4629839

[5] Dixon BP, Hulbert JC, Bissler JJ. Tuberous sclerosis complex renal disease. Nephron Exp Nephrol 2011; 118:e15–20. doi: 10.1159/000320891 21071977 10.1159/000320891 PMC2992644

[6] Bissler JJ, Kingswood JC, Radzikowska E, Zonnenberg BA, Frost M, Belousova E, et al Everolimus for renal angiomyolipoma in patients with tuberous sclerosis complex or sporadic lymphangioleiomyomatosis: extension of a randomized controlled trial. Nephrol Dial Transplant 2016; 31:111–119. doi: 10.1093/ndt/gfv249 26156073 10.1093/ndt/gfv249

[7] Bissler JJ, Kingswood JC, Radzikowska E, Zonnenberg BA, Frost M, Belousova E, et al Everolimus for angiomyolipoma associated with tuberous sclerosis complex or sporadic lymphangioleiomyomatosis (EXIST-2): a multicentre, randomised, double-blind, placebo-controlled trial. Lancet 2013; 381:817–824. doi: 10.1016/S0140-6736(12)61767-X 23312829 10.1016/S0140-6736(12)61767-X

[8] Bissler JJ, Kingswood JC, Radzikowska E, Zonnenberg BA, Belousova E, Frost MD, et. al Everolimus long-term use in patients with tuberous sclerosis complex: Four-year update of the EXIST-2 study. PLoS One. 2017 8 9;12(8):e0180939 doi: 10.1371/journal.pone.0180939 28792952 10.1371/journal.pone.0180939 PMC5549893

[9] Williams JM, Racadio JM, Johnson ND, Donnelly LF, Bissler JJ. Embolization of renal angiomyolipomata in patients with tuberous sclerosis complex. Am J Kidney Dis 2006; 47:95–102. doi: 10.1053/j.ajkd.2005.09.028 16377390 10.1053/j.ajkd.2005.09.028

[10] Eijkemans MJ, van der Wal W, Reijnders LJ, Roes KC, van Waalwijk van Doorn-Khosrovani SB, Pelletier C, et al Long-term Follow-up Assessing Renal Angiomyolipoma Treatment Patterns, Morbidity, and Mortality: An Observational Study in Tuberous Sclerosis Complex Patients in the Netherlands. Am J Kidney Dis 2015; 66:638–645. doi: 10.1053/j.ajkd.2015.05.016 26165440 10.1053/j.ajkd.2015.05.016

[11] Jinzaki M, Tanimoto A, Narimatsu Y, Ohkuma K, Kurata T, Shinmoto H, et al Angiomyolipoma: imaging findings in lesions with minimal fat. Radiology 1997; 205:497–502. doi: 10.1148/radiology.205.2.9356635 9356635 10.1148/radiology.205.2.9356635

[12] Hafron J, Fogarty JD, Hoenig DM, Li M, Berkenblit R, Ghavamian R. Imaging characteristics of minimal fat renal angiomyolipoma with histologic correlations. Urology 2005; 66:1155–1159. doi: 10.1016/j.urology.2005.06.119 16360431 10.1016/j.urology.2005.06.119

[13] Lane HA, Wood JM, McSheehy PM, Allegrini PR, Boulay A, Brueggen J, et al mTOR inhibitor RAD001 (everolimus) has antiangiogenic/vascular properties distinct from a VEGFR tyrosine kinase inhibitor. Clin Cancer Res 2009; 15:1612–1622. doi: 10.1158/1078-0432.CCR-08-2057 19223496 10.1158/1078-0432.CCR-08-2057

[14] McCormack FX, Inoue Y, Moss J, Singer LG, Strange C, Nakata K, et al Efficacy and safety of sirolimus in lymphangioleiomyomatosis. N Engl J Med 2011; 364:1595–1606. doi: 10.1056/NEJMoa1100391 21410393 10.1056/NEJMoa1100391 PMC3118601

[15] Dabora SL, Franz DN, Ashwal S, Sagalowsky A, DiMario FJ Jr, Miles D, et al Multicenter phase 2 trial of sirolimus for tuberous sclerosis: kidney angiomyolipomas and other tumors regress and VEGF- D levels decrease. PLoS One 2011; 6:e23379 doi: 10.1371/journal.pone.0023379 21915260 10.1371/journal.pone.0023379 PMC3167813

